# Persistent post-traumatic headache: a migrainous loop or not? The clinical evidence

**DOI:** 10.1186/s10194-020-01122-5

**Published:** 2020-05-24

**Authors:** Alejandro Labastida-Ramírez, Silvia Benemei, Maria Albanese, Antonina D’Amico, Giovanni Grillo, Oxana Grosu, Devrimsel Harika Ertem, Jasper Mecklenburg, Elena Petrovna Fedorova, Pavel Řehulka, Francesca Schiano di Cola, Javier Trigo Lopez, Nina Vashchenko, Antoinette MaassenVanDenBrink, Paolo Martelletti, On behalf of the European Headache Federation School of Advanced Studies (EHF-SAS)

**Affiliations:** 1 Division of Vascular Medicine and Pharmacology, Erasmus University Medical Center, Rotterdam, The Netherlands; 2 Health Sciences Department, University of Florence, and Headache Centre, Careggi University Hospital, Florence, Italy; 3 Department of Systems Medicine, Neurology Unit, University of Rome “Tor Vergata”, “Tor Vergata” Hospital, Rome, Italy; 4 Department of Child Neuropsychiatry, University of Palermo, Palermo, Italy; 5 Department of Child Neuropsychiatry, A.R.N.A.S. Civico, P.O. Giovanni di Cristina Ospedale dei Bambini, Palermo, Italy; 6 Diomid Gherman Institute of Neurology and Neurosurgery, Headache Centre and Nicolae Testemițanu State University of Medicine and Pharmacy, Chișinău, Republic of Moldova; 7 Department of Neurology, University of Health Sciences, Sisli Hamidiye Etfal Training and Research Hospital, Istanbul, Turkey; 8 Department of Neurology, Charité Universitätsmedizin Berlin, Berlin, Germany; 9 Department of Neurology, Zdorovie Clinic, Tomsk, Russia; 10 Department of Neurology, St. Anne’s University Hospital and Faculty of Medicine, Masaryk University, Brno, Czech Republic; 11 Neurology Unit, Department of Clinical and Experimental Sciences, University of Brescia, Brescia, Italy; 12 Department of Neurology, Hospital Clínico Universitario de Valladolid, Valladolid, Spain; 13 University Clinic of Nervous Diseases, Sechenov First Moscow State Medical University, Moscow, Russian Federation; 14 Department of Clinical and Molecular Medicine, Sapienza University of Rome, Rome, Italy

**Keywords:** Headache, Migraine, Trauma, Traumatic brain injury, Treatment

## Abstract

**Background:**

Headache is a common complication of traumatic brain injury. The International Headache Society defines post-traumatic headache as a secondary headache attributed to trauma or injury to the head that develops within seven days following trauma. Acute post-traumatic headache resolves after 3 months, but persistent post-traumatic headache usually lasts much longer and accounts for 4% of all secondary headache disorders.

**Main body:**

The clinical features of post-traumatic headache after traumatic brain injury resemble various types of primary headaches and the most frequent are migraine-like or tension-type-like phenotypes. The neuroimaging studies that have compared persistent post-traumatic headache and migraine found different structural and functional brain changes, although migraine and post-traumatic headache may be clinically similar. Therapy of various clinical phenotypes of post-traumatic headache almost entirely mirrors the therapy of the corresponding primary headache and are currently based on expert opinion rather than scientific evidence. Pharmacologic therapies include both abortive and prophylactic agents with prophylaxis targeting comorbidities, especially impaired sleep and post-traumatic disorder. There are also effective options for non-pharmacologic therapy of post-traumatic headache, including cognitive-behavioral approaches, onabotulinum toxin injections, life-style considerations, etc.

**Conclusion:**

Notwithstanding some phenotypic similarities, persistent post-traumatic headache after traumatic brain injury, is considered a separate phenomenon from migraine but available data is inconclusive. High-quality studies are further required to investigate the pathophysiological mechanisms of this secondary headache, in order to identify new targets for treatment and to prevent disability.

## Background

Traumatic brain injury (TBI) results from an external mechanical force to the brain that usually leads to acute or persistent headache, which is one of the most disabling sequelae following trauma. According to the International Classification of Headache Disorders (ICHD-III), post-traumatic headache (PTH) is a secondary headache attributed to trauma or injury to the head that develops within 7 days: (i) following trauma, (ii) after regaining consciousness and/or (iii) after recovering the ability to sense and report pain [1]. Acute PTH is defined when the headache resolves within 3 months after TBI, whereas persistent PTH is characterized by headache lasting longer than 3 months.

Persistent PTH is frequent after mild TBI [2], accounting for 4% of all secondary headache disorders [3]. It is estimated that 14 to 58% of patients with TBI will develop a headache at 1 year after the trauma [4, 5] as shown in Table 1. The one-year prevalence of persistent PTH has been estimated 0.21% in Norway [6], whereas the lifetime prevalence of PTH in Denmark is 4.7% in men and 2.4% in women [7]. In a large cohort during the first year after TBI, the incidence of new-onset headache was 44% and the cumulative incidence of headache at 12 months was 71%, with a 20% incidence of persistent PTH [8].

**Table 1:** Comparison of the characteristics between persistent PTH and primary headaches

	Persistent PTH	Migraine	Tension-type headache	Cluster headache
**Prevalence**	18–58% after TBI	6–33%	62%	0.1%
**Risk factors**	-Prior history of headache -Female gender - Older age - Family history of headache	-Young age -Female gender	-Anxiety -Depression	-Young age -Male gender
**Duration of episodes**	Variable	180 min-3 days	30 min-7 days	15–180 min
**Headache symptoms**	-Migraine-like -Tension-type headache like -Cluster like	-Severe intensity -Unilateral location -Pulsatile quality -Aggravated by activity	-Mild/moderate intensity -Bilateral location -Pressing quality -Not aggravated by activity	-Severe intensity -Unilateral, orbital or periorbital
**Associated symptoms**	-Sleep disorders -Affective and behavioral disorders -Cognitive deficits	-Nausea or vomiting -Photophobia and phonophobia	-Photophobia, phonophobia or nausea	-Conjunctival injection, nasal congestion, eyelid edema, miosis, ptosis. -Sense of restlessness or agitation
**Imaging (MRI)**	-Less cortical thickness in bilateral frontal regions and right hemisphere parietal regions of the brain -Gray matter changes in the prefrontal cortex.	-White matter hyperintensities	-Normal	-Normal
**Neurophysiological studies (EEG)**	Early abnormalities (focal slowing, absence of activity, amplitude asymmetries)	-H response to flicker stimulation -Abnormal resting-state EEG rhythmic activity	Normal	Normal
**Treatment**	-Behavioral -Drugs depending on phenotype	-Acute: NAIDs / triptans -Preventive: β-blockers, antiepileptics, antihypertensive, CGRP Abs	-Acute: NAIDs -Preventive: antidepressants	-Acute: triptans/O2 -Preventive: corticosteroids, verapamil

Potential risk factors for developing persistent PTH include female gender, older age, presence of headache at the emergency room and pre-existing headaches [9–11]. Migraine patients who developed PTH have a 2-fold increase in the frequency and/or intensity of the headache after the injury [8, 12], whereas PTH patients with pre-existing tension-type headache also experience a slight increase in attack frequency [5, 13]. Moreover, patients with a family history of primary headache disorders have an increase probability to develop PTH [14, 15]. In addition, various surgical procedures in the craniofacial region, such as a craniotomy followed by meningeal irritation, have been attributed to cause different PTH phenotypes [16], however, the available data are very limited and the underlying pathogenesis remains to be stablished.

In contrast, the severity of headache, recovery time, race, marital status, level of education, alcohol use at the time of injury, the etiology of TBI and Glasgow Coma Scale scores are not risk factors for the development of persistent PTH [9, 10, 17]. Although phenotypic similarities between migraine and PTH have been demonstrated in previous studies, i.e. throbbing headache, pain on one side of the head, headache exacerbated by physical activities, moderate to severe pain, headache accompanied by nausea or vomiting, and photophobia and/or phonophobia [18–22], the exact relationship between persistent PTH and migraine remain largely unknown and the shared pathophysiological mechanisms are conflicting [23]. In a neuroimaging study, Chong et al. found that there were significant differences in fiber tract profiles between patients with migraine and persistent PTH in the bilateral anterior thalamic radiations, cingulum, longitudinal fasciculi, and uncinate fasciculi [24]. Moreover, it has been shown that regional brain volumes and cortical thickness in patients with persistent PTH with migrainous features and migraineurs revealed differences in measurements of brain volume and/or thickness [25].

Thus, the aim of this review is to discuss if there is a migrainous loop for persistent PTH and to provide a better understanding of the underlying mechanisms of migraine and headache attributed to TBI according to current evidence.

## Clinical presentation

Even though there are no major clinical features for persistent PTH, its clinical presentation is usually characterized by heterogeneous symptoms such as nausea, vomiting, headache after physical activity and stress, headache exacerbated by light and sound, and impaired cognitive and psychosocial functions [18]. These symptoms resemble those of various types of primary headaches, and the most frequent PTH phenotypes are migraine-like or tension-type-like headache [4, 14]. Some studies report that tension-type-like headaches are the most frequent [26, 27], while others show a higher prevalence of migraine- like headache in patients with PTH [5, 22]. In one study, for instance, tension-type-like headache was reported in 97% of patients with de novo headache after mild TBI and persistent PTH [27]. In another study, a migraine-like headache or a probable migraine-like headache was described in 49% of patients with persistent PTH after mild TBI [5]. Even two other studies [8, 13], showed migraine-like or probable migraine-like headache in 53% of patients with headache after moderate to severe TBI. Patients with migraine-like PTH are more likely to have headache several days a week or daily [13]; these patients have a higher probability to have migraine 1 year after injury [5]. As mentioned above, several studies have reported that individuals with persistent PTH develop migrainous features, including the throbbing headache, unilateral location, pain exacerbation by physical activities, moderate to severe pain, headache accompanied by nausea or vomiting, and photophobia and/or phonophobia [18–22]. However, in some cases headache may be the only clinical manifestation of PTH or it might be associated with cognitive and psychological symptoms [28]. According to the data reported in literature [27, 29], patients with persistent PTH may also have post-traumatic stress disorder or symptoms of anxiety and depression [11, 30]. It seems that anxiety and depression are more common among patients with persistent PTH than among migraineurs or healthy controls [25]. In addition, patients with PTH might suffer from autonomic dysfunction symptoms; the most common autonomic symptoms are orthostatic intolerance and bladder incontinence [31].

Less common PTH phenotypes are those similar to trigeminal autonomic cephalalgias: hemicrania continua-like [32, 33], chronic paroxysmal hemicrania-like [32, 34], cluster-like headache [32, 35] or short-lasting unilateral neuralgiform headache attacks with conjunctival injection and tearing (SUNCT). Patients with PTH might simultaneously fulfill criteria for other secondary headaches - e.g. cervicogenic headache [36] or medication overuse headache (MOH) [26, 27].

However, existing descriptive data concerning PTH should be interpreted with caution due to substantial methodological differences among studies [14, 26, 27]. Firstly, different inclusion criteria for (persistent) PTH have been established in some studies, potentially weakening evidence of causation - for example longer interval between trauma and headache development [26, 27]. Typically, in prospective studies subjects were enrolled within 1 week after head injury, while not in those retrospectives, possibly due to difficulties to obtain reliable information from the patients’ medical records.

Another potential pitfall represents the inclusion of patients with pre-existing headaches, as these individuals are unlikely to represent the PTH prevalence rates of the general population. If pre-existing headache patients are included, the different clinical headache type onset rather than simple worsening of the same type of headache should be required [26]. Further reason for discrepancies among studies might be the enrollment of patients with moderate or severe TBI, as there is as inverse relationship between the severity of TBI and PTH occurrence, with higher PTH rates in patients with mild TBI [26]. Moreover, about one third of patients suffering from PTH display multiple head pain phenotypes [27].

Interesting data concerning the co-occurrence of PTH and MOH have been provided by the Danish Headache Center, a tertiary headache center. In a study conducted between January 2001 and June 2003 among 53 patients with persistent PTH, analgesic overuse was identified in 42% [26]. In a following study realized between June 2008 and August 2011, among 90 patients with persistent PTH, only 13% of patients have had a MOH history during the PTH [27]. As suggested by the authors, these results are not directly comparable due to different sample selections, but it might reflect an increased patient awareness of the risk of developing MOH [27].

In conclusion, there are different variables which might play a role in the assesment of patients with persistent PTH. However, currently, the close temporal relation between trauma and headache onset is a very important element to make an appropriate diagnosis of PTH.

## Neuroimaging studies

Despite the high prevalence of PTH, there is still no clear understanding of the pathophysiology of this headache. The overlap of symptoms between persistent PTH and migraine forces to speculate about a common pathogenesis of these diseases, therefore, it could be very useful to conduct neuroimaging research in this area. Modern imaging techniques such as advanced magnetic resonance imaging (MRI), diffusion tensor imaging (DTI) and cerebral blood flow (CBF) measurements are useful to assess brain changes even in the absence of significant structural damages in mild TBI, which allows us to use them to study PTH.

Neuroimaging studies in persistent PTH have been performed to establish the brain structural [37–41] and functional changes [42–45] that could explain its pathophysiology, these include structural changes of the brain volume and density by voxel-based morphometry [25, 37] and brain thickness [38]. The white matter changes were studied by DTI measuring decreased fractional anisotropy, mean diffusivity and radial diffusivity and establishing the fiber tract profiles [24, 39]. The gray matter changes in persistent PTH were measured by gray matter volume [40] and voxel-based morphometry [37]. The functional changes in the brain associated with persistent PTH were studied using default mode network [42], resting state MRI with static and dynamic functional connectivity [43], perfusion weighted imaging [46], CBF [45] and proton spectroscopy [44]. Some studies compare persistent PTH with healthy controls [25, 37–45] and others with migraine patients [24, 25, 43, 45]. The studies that have compared persistent PTH with migraine, found structural [25, 38] and functional brain changes [43] between groups, which suggest that migraine and persistent PTH have different pathophysiologic mechanisms.

For example, Niu et al. conducted an MRI resting-state functional connectivity study in 54 patients with mild TBI (without pre-existing headaches) who developed acute PTH at 1- and 12-weeks following concussion [42]. Patients with PTH at 12-weeks documented a weaker baseline connectivity between the periaqueductal gray, a brain area involved in opioid antinociception, and the right inferior parietal lobule, a region of the default mode network involved in introception and self-reference [42]. The study concluded that disrupted periaqueductal gray – default mode network functional connectivity may be used as an early imaging biomarker to identify patients at risk of developing PTH [42].

Moreover, a study conducted by Obermann et al. in 32 patients with whiplash injury without pre-existing headaches, found that patients who developed persistent PTH, compared to healthy controls and patients affected by acute PTH, showed a decreased anterior cingulate and dorsolateral prefrontal cortex (DLPFC) gray matter density, areas of the default mode and salience network [37]. At an additional 12 months follow-up such differences were not present anymore, but an increased gray matter density in areas of the midbrain, thalami and cerebellum following persistent PTH resolution [37]. Arguably, the latter finding might represent adaptive changes to chronic pain processing [37]. These two studies demonstrated the importance of gray matter structural and functional changes over time in persistent PTH.

Various studies compared neuroimaging advanced measures of brain structure between persistent PTH, mild TBI without headache and healthy controls. Relative to healthy controls, PTH patients have been found to display reduced cortical thickness in various bilateral frontal and right parietal regions, with headache burden being negatively correlated to bilateral superior frontal cortex thickness [38]. Relative to mild TBI patients who do not developed headache, PTH patients had decreased gray matter volumes within two large clusters described as the right anterior-parietal and the left temporal-opercular [40], areas previously reported to be involved in both migraine and chronic pain [25].

Being migraine the usually clinical phenotype of (persistent) PTH, it is not surprising that different authors have investigated differences/overlaps between these two conditions in order to delineate whether there might be a common pathophysiology. Schwedt et al. compared measures of brain regional volumes, cortical thickness, surface area and brain curvature amongst twenty-eight: migraine patients, individuals with persistent PTH following a TBI without history of pre-existing headaches and healthy controls [25]. The authors found significant differences in brain structures between persistent PTH and migraine patients – namely the left precuneus, left caudal middle frontal lobe, left superior frontal lobe, right lateral orbitofrontal lobe and right supramarginal gyrus – but only the last three regions were found to be different between persistent PTH and healthy controls and none between migraine patients and healthy controls. This finding suggests a certain degree of brain structures involvement/pathophysiological specificity unique to persistent PTH regardless of the clinical phenotype [25].

A recent study by Dumkrieger et al. [43] investigated static and dynamic functional connectivity differences between 44 patients with persistent PTH attributed to mild TBI and 33 patients with migraine. Individuals with pre-existing headaches were excluded, with the exception of infrequent episodic tension-type headaches. Significant differences in static functional connectivity between migraine and persistent PTH were found in up to 17 region pairs that included the primary and secondary somatosensory cortex, insula, hypothalamus, anterior cingulate, precuneus, ventromedial prefrontal and DLPFC. Significant differences in dynamic functional connectivity between migraine and PTH were found in 10 region pairs that included the secondary somatosensory cortex, hypothalamus, middle cingulate, temporal pole, superior parietal and parieto-occipital cortex, cingulate and the amygdala. The majority of the afore mentioned brain areas are known to be involved in pain and visual-processing. After controlling for sex and age, there were significant correlations between years lived with headache with static functional connectivity of right primary somatosensory with left supramarginal gyrus and between headache frequency with static functional connectivity of the cingulate-insula-hypothalamus network in PTH patients [43]. This study demonstrates that persistent PTH patients have different static and dynamic functional connectivity compared with migraine patients for regions involved in pain processing.

Gilkey et al. were among the first to demonstrate the presence of structural brain changes in patients with persistent PTH (with unknown pre-existing headache history) attributed to mild TBI. In their pilot study, they compared the regional CBF (rCBF) of 35 patients with persistent PTH with minor TBI to 92 migraineurs and 49 healthy controls using the xenon 133 Xe inhalation rCBF technique [45]. Compared to migraine patients and healthy controls, patients with persistent PTH had significantly reduced rCBF and greater regional and hemispheric CBF asymmetries [45]. The authors suggested that changes in CBF and vasomotor instability with localized vascular hypersensitivity plays a pivotal role in the pathogenesis of persistent PTH and other symptoms associated with mild TBI [45].

Alhilali et al. compared DTI studies of 58 patients with acute or persistent PTH of a migraine phenotype with unknown pre-existing headache history, with 17 patients with mild TBI without headache [39]. It was found that patients with headache had decreased fractional anisotropy in the corpus callosum and fornix/septohippocampal circuit [39]. In contrast to the above studies, this observation allows us to consider the possibility of a common pathophysiological mechanism between persistent PTH and migraine, since white matter changes in the corpus callosum have also been identified in patients with migraine [39].

On the other hand, Chong et al. [24] demonstrated differences of white matter changes in patients with PTH without history of pre-existing headache, compared to migraine patients. In this DTI study the authors compared 49 patients with persistent PTH attributed to mild TBI to 41 patients with migraine and 41 healthy controls [24]. Node-by-node diffusion parameters (mean diffusivity and radial diffusivity) were calculated per groups. A significant difference of mean diffusivity or radial diffusivity in the bilateral anterior thalamic radiations, cingulum (angular bundles and cingulate gyri), inferior longitudinal fasciculi, and uncinate fasciculi, the left corticospinal tract, and the right superior longitudinal fasciculi-parietal portion was revealed between patients with persistent PTH and migraine patients. Also, in patients with PTH a positive correlation was found between headache frequency and cingulate angular bundle diffusion parameters, which were not observed in patients with migraine. The authors concluded that disease-specific differences between groups might suggest different pathophysiological mechanisms underlying these conditions [24].

As follows from the above listed facts, there is more evidence of brain structural differences in migraine patients and patients with persistent PTH, which allows us to consider these types of headache as distinct nosological entities. However, high-quality studies are further required to investigate the structural brain changes specific to PTH.

## Neurophysiological studies

### Electroencephalography (EEG)

Electroencephalographic studies in PTH are sparse and the few available often show marked early abnormalities, these include focal slowing, absence of fast activity and amplitude asymmetries [47–49]. Torres and Shapiro reported abnormalities in 44% of patients of a group with mild closed head injuries compared to a group with whiplash injury alone [48]. A more recent study on patients with mild or minor closed head injuries, selected on the basis of of lack of unconsciousness on admission and normal neurological examinations, revealed 54% with focal and diffuse slowing EEG abnormalities [49].

The EEG may be abnormal immediately after injury, but it often normalizes within minutes to weeks. Persistent findings that were once considered abnormal are now considered normal variants, having the same incidence as in the general population [50]. Quantitative EEG may or may not be useful in head injury. In one small study, quantitative EEG showed a statistically significant increase in both slow and fast activities over the temporal region of the skull [51]. However, the authors concluded that this test offers little benefit to the patient with PTH, since there is a great variability within both PTH and healthy controls [51]. Another study examined the ability of power spectrum analysis to discriminate between 608 head injury patients and 108-age matched controls with more than 90% accuracy [52]. No correlation was made between the symptoms of PTH or post-traumatic syndrome and abnormal test results [52]. Therefore, the predictive value of this analysis is uncertain at this time. In general, these studies indicate that PTH patients, as a group, differ from non-headache controls; but unlike the study of Ramadan et al. [53], they cannot reliably differentiate an individual PTH patient from an idiopathic headache patient.

Contrary to what has been observed in PTH patients, several previous EEG studies emphasized abnormal electrocortical activities in migraine patients. The most frequently described electrocortical phenomena in migraineurs were the so-called H response to flicker stimulation – also known as enhanced photic driving (PD) – and the abnormal resting-state EEG rhythmic activity [54]. Enhanced PD of EEG during intermittent photic stimulation using fast Fourier transform analysis on steady-state visual evoked potentials (VEP), the so-called H response, was more prevalent in migraine patients than in healthy controls. Researchers observed that the fundamental components of the EEG spectra were increased equally in both migraine with aura (MA) and migraine without aura (MO) patients [55], predominantly in the temporo-parietal regions, with reduced interhemispheric coherence in fronto-temporo-parietal areas [54, 55]. De Tommaso et al. observed that, although in both MO and MA groups PD was significantly enhanced with respect to controls, those patients experiencing aura showed more pronounced decreased phase synchronization between beta rhythms and higher Granger causality values – measuring the flow of connections and information across different brain areas – during light stimulation compared to MO patients [56]. During the interictal period of MA patients, the quantitative analysis of spontaneous electroencephalographic activity showed alpha rhythm and peak frequency asymmetries over the posterior regions, increased power of alpha rhythm, and widespread increase in delta and theta total power in comparison with healthy controls [54]. Reduction of alpha rhythm or unilateral reduction of alpha and theta activity was detected in MA patients with a pure visual aura, mostly contralateral to the neurological signs [57]. MA patients had greater alpha peak power interhemispheric asymmetry, chiefly in the posterior regions, and unrelated to the headache side, than MO [54]. In conclusion, EEG studies confirm that despite significant overlap in symptoms between PTH and migraine, there are identifiable neurophysiological differences in brain function, perhaps suggesting distinct underlying pathophysiology between the two headache types.

### Evoked potentials

Short-latency somatosensory evoked potentials have not been shown to have an important value in testing patients with head injury or post-traumatic headache [12]. Brain-stem auditory evoked potentials have been found to be abnormal in 10% to 20% of patients with head injury and post-concussion syndrome, more frequently in those with prolonged unconsciousness [50]. They can either improve or deteriorate from 2 days to 1 month after injury [58]. Symptomatic dizziness does not correlate with brainstem auditory evoked potential abnormalities [58]. While the brainstem auditory evoked potential separates groups of PTH patients from groups of controls, it is of no value in distinguishing an individual with post-traumatic syndrome from one without it.

On the other hand, higher cortical response amplitudes, an increased interhemispheric response asymmetry, and a deficit of response amplitude decrement were demonstrated by using different types of sensory stimuli and techniques in most MA patients [54]. Because in most cases the aura is visual, the major part of the published studies investigated VEPs to search for cerebral signatures associated with MA. By analyzing the evoked responses in a classical way of averaging a large quantity of trials, mainly increased amplitudes of steady-state (SS) or transient VEPs have been discovered in MA patients during attack-free intervals [54]. In some reports the grand-average of VEP N75-P100 and/or P100-N145 amplitudes has been found greater in MA patients than in controls and/or in MO patients [54, 59, 60]. In addition, decreased amplitude of the prerolandic component (N20) of somatosensory evoked potentials in both MO and MA patients has been found in one study [61], but amplitudes were within the normal range in other [62]. Most of the researchers who recorded short-latency brainstem auditory evoked potentials were not able to find any interictal abnormalities in migraine, probably because they pooled patients with different migraine phenotypes (MO and MA or different MA subtypes) in different proportions in a single group [63].

The role of P300, an event-related potential, in evaluating PTH patients is also uncertain up to date. One study demonstrated significant abnormalities of P300 amplitude and latency in 20 head injury patients compared with 20 control subjects [64]. Werner and Vanderzant found an abnormal response in only one of 18 patients [65]. Kobylarz et al. reported a correlation between an abnormal P300 and an abnormal MRI [66]. An interesting study demonstrated that hearing accident-related words (i.e., “stressful”) produced a significantly larger P300 than hearing neutral words in patients with mild head injury but not in non-head injury controls. The P300 amplitude difference correlated with the patient’s two-way state anxiety score [67].

Nevertheless, enough studies suggested that basic P300 amplitude tended to be the greatest in a mixed group of MO and MA patients compared with controls and other types of headaches [54]. P300 amplitude was significantly reduced during mind wandering relative to on-task periods in migraineurs, contrasting to what happened in healthy controls [54, 68]. The authors argued that a more consistent propensity towards engaging in response attenuation during mind wandering states may provide migraineurs with an alternative compensatory strategy for reducing stimulus overload in cortex [68].

Electronystagmogram is abnormal in 40–50% of patients with head injury or “whiplash” in clinic-based studies. Toglia examined 150 patients who complained of vestibular symptoms following either head injury or whiplash injury, searching for spontaneous, latent, and positional nystagmus [69]. Bithermal caloric tests and rotational tests were performed when possible. Abnormal caloric tests (including both canal paresis and directional preponderance) were found in 63% of head injury patients. Abnormal rotatory tests were found in 9 of 16 whiplash patients (56%) and 20 of 24 head injury patients (83%) [69]. Rowe and Carlson studied 19 patients with post-concussive dizziness following head injury and found that 11 patients (58%) had abnormalities consisting of latent or positional nystagmus or calorie-induced nystagmus [70]. None of these patients, who had abnormal brainstem auditory evoked potentials (3 standard deviations), showed a normal electronystagmogram. Conversely, most patients with abnormal electronystagmograms had normal brainstem auditory evoked potentials [70]. This suggests that the electronystagmogram may be more sensitive than the brainstem auditory evoked potential. Mallinson and Longridge found electronystagmogram abnormalities in patients with whiplash injury and mild head injury, but not in whiplash injury alone, although symptoms of dizziness were similar in both groups [71]. Computerized dynamic posturography was studied in a referral population of dizzy patients after whiplash injury, alone or with mild head injury. Both groups had positive findings, although the type of posturography abnormality was different between groups [71]. To summarize, evoked potentials can also be used to detect post-traumatic neurophysiological changes in PTH patients, but they are relatively subtle and may be challenging to distinguish from those seen in other conditions such as migraine.

## Treatment

As previously discussed, persistent PTH can resemble the clinical features of migraine, tension-type headache or other primary headache disorders, but the signs and symptoms are often mixed in nature and can be challenging to manage. Also, the impact of external factors such as psychosocial and legal circumstances surrounding the injury must be taken into account, since post-traumatic stress disorder is known to co-occur and affect the intensity of persistent PTH [72, 73]. Currently, there is no available data from randomized controlled trials evaluating the therapeutic efficacy of medical interventions specific to persistent PTH, therefore, the therapy mirrors conventional treatment approaches for non-traumatic primary headache disorders [74]. Many experts agree that persistent PTH should be treated “according to the class of headache its characteristics most closely resemble” [3, 18, 75]. However, this approach lacks evidence and often results in poor treatment responses [76]. It is also important to use personalized medicine which requires a clinical approach integrated with the use of pharmacological and physiotherapeutic strategies as well as educational and behavioral interventions, often combined among them, because the multidisciplinary approach to treatment is likely to be most efficacious in the treatment of persistent PTH [77].

The selection of an appropriate pharmacologic therapy for treating persistent PTH includes consideration of abortive medications that stop the acute pain attack, and often a prophylactic medication that focuses on decreasing the attack frequency. The choice of particular agents depends on their efficacy for the primary headache it is resembling, side-effect profiles, and patient comorbidities [78]. Since we believe that persistent PTH is not a migrainous loop, and it has been shown that it often mimics a migraine-like or tension-type headache-like phenotype [79], we discuss persistent PTH treatments based on the different headache presentations.

### Pharmacological treatment of persistent PTH

#### Persistent PTH resembling tension-type headache (TTH)

Standard pharmacologic interventions for TTH include over-the-counter preparations, non-steroidal anti-inflammatory drugs and rarely opioids. Regardless of the agent employed, successful headache treatment is most likely if the medication is taken at the onset of a headache rather than waiting for the headache pain to escalate. Common over-the-counter medications trialed by patients include acetaminophen (paracetamol), aspirin, ibuprofen, and naproxen, all of which may come in combination with caffeine. Opioid medications are rarely indicated to treat a severe refractory headache; caution must be used, however, as repeated use of opioids can lead to addiction and, similar to other analgesics, MOH, therefore they should be avoided in the treatment of PTH [80–82].

As discussed above, persistent PTH resembling chronic tension-type headache is one of the most or the most common headache type presentation in studies performed in PTH [4, 26, 27, 83]. Patients with chronic or very high-frequency TTH are in need of prophylactic therapies [84, 85]. Treatment is primarily based on the treatment of chronic tension-type headache. Retrospective studies in populations with persistent PTH showed that prophylactic medication with amitriptyline is an effective treatment [26, 86]. Experts in the field also recommend the use of nortriptyline, the metabolite of amitriptyline, which shows better tolerability due to lower anticholinergic side effects [87].

#### Persistent PTH resembling migraine

In migraine, potential abortive agents include the same medications as in tension-type headache, but also triptans, and, less commonly, ergot derivatives or opioids among others. Triptans are selective serotonin receptor agonists acting at the 5-HT_1B/1D/(1F)_ receptors present in the trigeminovascular system. These agents have shown a well-defined efficacy in multiple clinical trials and hold a level A recommendation for use in the abortive treatment of migraine [79]. Other treatment options for migrainous headaches includes antiemetic agents. Agents such as metoclopramide, promethazine, and prochlorperazine may also be tested in patients that are refractory to specific migraine treatments. A potential advantage to the use of these medications is the lack of risk of rebound headache [88].

When the decision has been made to initiate prophylactic therapy for PTH, the selection of a specific agent usually depends on the comorbidities (i.e., amitriptyline should be considered if the patients have concomitant insomnia or β-blockers if concomitant hypertension) and contraindications (β -blockers/Calcium-channel blockers should be avoided if the patient has arterial hypotension, tricyclic antidepressants in the case of excessive fatigue, QT prolongation, increased ocular tone, etc.) [89]. There are many classes of drugs, which have been employed for the prophylaxis of migrainous headaches.

Prophylactic choices include β-blockers (which have particular utility in patients with anxiety as they may decrease the physical autonomic symptoms of anxiety); tricyclic antidepressants, which are particularly effective in patients with depression or sleep disturbances; calcium channel blockers, valproic acid, topiramate, gabapentin, and onabotulinum toxin A. However, in a study where PTHs were primarily resembling migraine, tricyclic antidepressants in low doses (25–50 mg of amitriptyline daily) were found to be not effective and the authors conclude that amitriptyline, when used, should be titrated up to higher doses to be effective [21].

There has been some exploration of naturopathic agents such as feverfew and butterbur, as well as supplements such as magnesium, riboflavin (vitamin B2) and coenzyme Q10. Magnesium may be effective as a prophylactic treatment with 400 mg daily of chelated magnesium, magnesium oxide or slow-release magnesium in patients with symptoms suggestive of hypomagnesemia, such as migraine headaches, premenstrual syndrome, cold extremities and leg or foot muscle cramps [90]. There are positive but small controlled trials for riboflavin [91] and coenzyme Q10 [92] and stronger evidence for butterbur [93]. Unfortunately, concerns have been raised regarding the preparation process for commercially available butterbur with potential for hepatotoxicity [94, 95].

#### Persistent PTH resembling trigeminal autonomic Cephalalgias (TACs)

There are few case reports of cluster headache occurring after trauma and most of these do not fulfill the seven-day criteria for PTH, but a correlation between head injuries and cluster headaches has been reported, although it is unclear if head trauma is causative for the development of cluster headache or if cluster headache is associated with a higher risk of head trauma [96]. In the cases with cluster headache following the incident within 7 days, headache always appeared on the side of the trauma and the treatment of choice are the same agents as in primary cluster headache, with sumatriptan, oxygen or intravenous dihydroergotamine [97] as abortive medication and verapamil as preventive agent [98].

There is a small number of case reports of successful treatment of PTH with indomethacin appearing as hemicrania continua and paroxysmal hemicrania [33, 34, 99]. There are two cases reported with persistent PTH appearing as Short-Lasting Unilateral Headache With Cranial Autonomic Symptoms (SUNA) showing successful treatment with gabapentin or carbamazepine [100].

#### Medication overuse headache (MOH)

There is a significant risk of MOH in the PTH population [101], with the use of over-the-counter and other analgesics leading to an overall increase in headache frequency. MOH in PTH often resembles the underlying headache type, therefore also appearing resembling TTH or migraine [102, 103]. Analgesic overuse was recorded in 19 to 42% of the study populations and a significant part of these patients improved after discontinuation of the overuse [26, 83]. Therefore, MOH should always be considered when assessing patients with persistent PTH and analgesic medication withdrawal is the treatment of choice.

#### Anti-CGRP monoclonal antibodies

Calcitonin Gene-Related Peptide (CGRP) is a potent endogenous vasodilator and neurotransmitter, which is involved in the pathophysiology of migraine and has been a target for drug development in recent years [104–106]. In experiments, it has been shown that the activation of the trigeminal ganglion leads to the release of CGRP [107]. Anti-CGRP monoclonal antibodies such as erenumab, eptinezumab, fremanezumab and galcanezumab have shown to be effective for preventive treatment of episodic and chronic migraine and are currently approved (erenumab, fremanezumab, galcanezumab) or are expected to seek approval (eptinezumab) from the European Commission for preventive treatment of episodic and chronic migraine [108–111]. As described above, the clinical appearance of PTH often resembles migraine, therefore it is commonly assumed that there may be a similar use case in the treatment of PTH with migraine-like features. Recent experiments in rodent models have shown CGRP involvement and efficacy of anti-CGRP monoclonal antibodies in PTH [112, 113]. Currently, there is only one observational clinical study completed, evaluating the treatment of PTH appearing as migraine phenotype with erenumab in 7 patients, which showed exceptional efficacy of 140 mg erenumab measured in reduction of headache days and Head Impact Test-6 [114]. Impressively, in most of these cases there was only one application of erenumab required for stable remission of the symptoms in the follow-up over 6 months, only in one patient the erenumab dose was administered twice. However, the small quantity of patients and the fact that three of them had pre-existing migraine, limits the generalization of these results. Hence, this work should be interpreted cautiously, since it is unlikely to reliably reflect data from the ongoing clinical trials with erenumab for PTH prevention.

On the publishing date of this review, there are two studies currently recruiting for treatment of PTH with erenumab (NCT03974360) and fremanezumab (NCT03347188), although only the study with fremanezumab is placebo-controlled [115, 116]. In conclusion, at the moment there is a high need for the clinical trial results, but there is growing evidence for the efficacy of anti-CGRP monoclonal antibodies in persistent PTH and they are likely to be a promising future treatment of persistent PTH with migraine phenotype.

#### Other treatment approaches

There are different techniques of injections with onabotulinum toxin A, local anesthetics and steroids currently used in the treatment of primary headache disorders and therefore also available in persistent PTH.

#### Onabotulinum toxin injections

At present, Botulinum Toxin (BTX) injection is the only FDA-approved medication for chronic migraine. There are a few case reports showing the efficacy of BTX in the treatment of persistent PTH. The most extensive study looked at the charts of 64 male servicemen age 20 to 50 who presented to the Concussion Care Clinic of Womack Army Medical Center in Fort Bragg North Carolina between 2008 and 2012 [117]. Of those patients, 36 (56.3%) had more than one type of headache, ten (15.6%) had more than two headaches, and 48 subjects (75%) had continuous headache. Forty-one patients (64%) reported that they were better after treatment, 18 (28%) were unchanged, two (3%) were worse, and three patients were lost to follow-up. Common side effects included headache and neck pain. In this author’s experience, these patients showed a significant improvement in their headaches and associated concussion symptoms after treatment with BTX.

#### Nerve blocks

Peripheral nerve blocks are one of the most widely used interventional procedures to treat persistent PTH [118]. Common sites include the greater occipital nerve, lesser occipital nerve, auriculotemporal nerve, supraorbital nerve, supratrochlear nerve, and sphenopalatine ganglion (SPG). Interventions include blocking a single nerve unilaterally, bilaterally or multiple nerves. The rationale being that local anesthetic to these nerves results in a decrease afferent feedback to the trigeminal nucleus caudalis, decreasing nociceptive transmission [119]. Typical anesthetics include bupivacaine (0.25 to 0.75%) or lidocaine (2%), with volumes ranging from 0.5 to 2 cc per site. Local anesthetics inhibit nerve fiber conduction by reversibly inhibiting sodium channels and can act on unmyelinated C-fibers and thinly myelinated Aδ fibers that mediate pain. Local anesthetics can be given alone, combined with each other and/or with a steroid. In a single-blind randomized controlled trial, Ashkenazi et al. compared the effect of lidocaine with triamcinolone vs. lidocaine alone in patients with transformed migraine [120]. No statistically significant differences were seen in any of the outcome measures between the two groups.

#### Trigger point (TP) injections

Common sites include the occipitalis, frontalis, masseter, temporalis, trapezius, levator scapulae, semispinalis capitis, splenius and sternocleidomastoid. The pathophysiological mechanisms underlying TPs are poorly understood. Therefore, in theory, amelioration of TP in the head and neck should result in a decrease in headache. As is the case with peripheral nerve blocks, TP injections can be performed with lidocaine and/or bupivacaine, but steroids are also often used. After location of the trigger point via palpation, multiple sites are often injected, 1 to 2 cc per site using a 0.5- to 1-in. needle. Unfortunately, there are no studies looking at the treatment of trigger points in PTH.

#### Non-drug treatments

Apart from drug-based therapies, several different non-drug treatments are currently used in the therapeutic approach in primary headaches, especially in migraine and TTH. Persistent PTH is often over-medicated with both prescription and over the counter pharmacotherapies [102, 121], therefore, a systematic management program that is headed toward reducing polypharmacy in persistent PTH patients can improve patient safety and reduce hospitalizations from the burden of headaches [122].

#### Physical medicine

There are very few studies that specifically look at physical therapy, massage therapy, spinal manipulation, and mobilization as a treatment for persistent PTH. In a case-control study [123], patients underwent physiotherapy by unblinded physical therapists with the primary endpoint being the change in headache intensity. The patients were randomly assigned to receive either manual therapy for the cervical region (usual care group) or additional manual therapy techniques to the temporomandibular region. Patients in the treatment group experienced statistically significant decreases in headache intensity at 3 and 6 months when compared to the usual care group. Two other modalities that have received considerable study are spinal manipulation and mobilization. When addressing the cervical spine, mobilization techniques are safer than manipulation techniques, which can be associated with adverse effects (i.e., disc herniation and arterial dissection).

Youssef et al. compared the efficacy of spinal mobilization with massage therapy in patients with cervicogenic headache [124]. Thirty-six subjects were randomized with eighteen receiving passive spinal mobilization for 30–40 min, and the other eighteen receiving massage therapy, myofascial release, traction, and stretching exercises. Both groups were treated for 12 sessions (2× per week for 6 weeks). Outcome measures included decrease in headache intensity, frequency, and duration, as well as improvement in cervical pain and range of motion. Both groups experienced significant improvements in all measured variables with the mobilization group experiencing statistically significant reductions in all variables when compared to the massage group [124]. Moreover, a case report by Channell et al. showed that a multidisciplinary approach, including medications and osteopathic manipulation, was effective in treating a 38-year-old female with chronic PTH [125].

#### Repetitive Transcranial magnetic stimulation (TMS)

TMS is a non-invasive neurostimulation procedure in which cerebral electrical activity is influenced by a pulsed magnetic field. An electric current briefly passing through a copper-wire coil generates the magnetic field. When this coil is placed on the head, its magnetic field induces small currents in an area of the brain directly under the coil. In repetitive TMS (rTMS), repeated single magnetic pulses of similar intensity are delivered over a targeted brain region.

One recent study by Leung et al. showed the benefits of rTMS treatment to the DLPFC in participants with mild TBI related headache [126]. They reported an average daily persistent headache intensity reduction at both one and 4 weeks following rTMS when compared to baseline. In addition, they found a significant reduction in the depression rating score at 1 week.

Similarly, Stilling et al. performed a double-blind, randomized, sham-controlled, pilot clinical trial on twenty participants (18–65 years) with persistent PTH and persistent post-concussion symptoms (PPCS) [127]. Ten sessions of rTMS therapy (10 Hz, 600 pulses) were delivered to the left DLPFC. The primary outcome was a change in headache frequency or severity at one-month post-rTMS. Two-week long daily headache diaries and clinical questionnaires assessing function, PPCS, cognition, quality of life, and mood were completed at baseline, post-treatment, and at one-, three-, and six-months post-rTMS. Secondary outcomes revealed an overall time interaction for headache impact, depression, post-concussion symptoms, and quality of life. This pilot study demonstrates an overall time effect on headache severity, functional impact, depression, PPCS, and quality of life following rTMS treatment in participants with persistent PTH, however, findings were below clinical significance thresholds. Since there was a 100% response rate, no dropouts, and minimal adverse effects, future larger studies are warrant.

#### Surgical decompression

Four different decompression procedures are performed based on the headache location, i.e., frontal, temporal, occipital, and sinus regions. The theory behind these procedures is that peripheral nerve compression in the head and neck can serve as a migraine trigger. Two studies have been published. The first study (placebo-controlled) enrolled 76 patients based on their response to BTX, with 49 receiving actual surgery and 26 receiving sham surgery [128]. The primary endpoint was a 50% reduction in migraine headache days. The baseline headache frequency of the subjects in the intervention group was 9.9 (±6.0) per month and 9.5 (±4.4) in the control group. At 1-year post surgery, 28 subjects reported complete resolution of their migraine headaches, with 41 reporting significant improvement. Of the 26 subjects who received sham surgery, one reported complete resolution of their migraine headaches, and 15 reported significant improvement.

The second study [129], a 5-year outcome study included 125 randomly assigned patients. The treatment group had a single surgery or a combination of procedures with follow-up at 1 and 5 years, while the placebo group received saline injections. Sixty-nine patients were eventually included in the final analysis with 9% having a single-site procedure, 22% having surgery at two sites, 44% at three sites, and 26% at four sites. Twenty subjects (29%) had complete resolution of their headaches, and 41 (59%) had significant improvement defined as a 50% reduction of frequency, intensity, or duration.

Interestingly, a recent retrospective review of 28 consecutive cases of patients with post-concussion headaches mirrors the above studies [130]. In this case, all patients had persistent PTH for at least 3 to 6 months and they underwent occipital nerve surgery including decompression or excision of the greater, lesser, or third occipital nerves. Those with headaches centered in the temple area underwent transection of the anterior branch of the auriculotemporal nerve or zygomaticotemporal nerve, and those with frontal headaches underwent decompression of the supraorbital, supratrochlear, and infratrochlear nerves. Preoperative and postoperative headache pain was evaluated with the visual analog scale (VAS) in 24 patients with at least 6-month follow-up. Of these patients, twenty-one (88%) had a successful outcome of at least a 50% reduction in their VAS following peripheral nerve surgery, and 12 patients (50%) were pain-free at the end of follow up, while eight patients required a second procedure, and four underwent a third procedure, which included readdressing the occipital region. The authors suggest that patients with concussion may experience a traction stretch injury to their peripheral nerves, which can then act as a potential pain generator.

There are numerous interventional procedures available for patients with acute, chronic, and refractory PTH, unfortunately, no prospective controlled trials exist and these are clearly needed. A multidisciplinary approach is preferred, therefore, in the acute phase, combining physical therapy with peripheral nerve blocks, trigger point injections and abortive medications is suggested, while in some cases preventative medications will more than likely yield the best outcome.

#### Intranasal block of the SPG

This intervention has been shown to be effective in the treatment of chronic migraine. A double-blind placebo-controlled study by Cady et al. looked at repetitive SPG blocks with 0.5% bupivacaine in chronic migraine treatment [131]. Thirty-eight patients received treatment twice a week for 6 weeks. Patients receiving bupivacaine reported on average 5.71 fewer headache days than placebo, a reduction in acute medication usage and improved quality of life measures.

A recent case report in sport-related PTH found similar efficacy in a patient having failed oral preventive measures [132]. After SPG block, this patient experienced resolution of headache and ultimately returned to school and sports without recurrence of symptoms during 6 months post-procedure.

#### Behavioral treatment

Martin et al. [133] developed a novel behavioral treatment approach for chronic primary headaches, referred to as “Learning to Cope with Triggers” (LCT). Triggers, most commonly stress, hormonal factors, light flicker or glare, noise, odors, certain foods, alcohol, weather changes, and fatigue often precede headaches. Health professionals and education materials typically advise avoidance of such triggers as good “headache hygiene.” However, avoidance behavior can become excessive, where pain-related fear and avoidance behavior contribute to disability in chronic headache sufferers. In response, Martin et al. designed a behavioral intervention that involves graded exposure to triggers. The rationale depends on the nature of the particular trigger. Graded exposure could serve as experiments to learn which alleged triggers do in fact reliably precipitate headaches, promote habituation, and/or provide opportunity to practice applying new coping skills. The intensity and frequency of exposures are progressively increased at a pace that is insufficient to provoke headaches, in collaboration with the patient. Avoidance is recommended over exposure for unhealthy triggers, such as dehydration, alcohol use, and inadequate sleep.

In a randomized controlled trial with patients who had chronic daily headaches of various types, LCT resulted in improved headache ratings and reduced medication use in comparison to avoidance coaching and wait-list control groups. The LCT approach may be especially well-suited to persisting PTH following mild TBI [134]. It has been recently shown that patients with persisting PTH after mild TBI identify a similar pattern of triggers as those in primary headache disorders, but perceive mental exertion as a particularly potent headache precipitant that they try to avoid. This “cogniphobia” is associated with headache frequency and intensity, and possibly reduced neuropsychological performance after mild TBI [135]. Cogniphobia could be easily added as a treatment target in LCT. There is also emerging evidence that an avoidant coping style is associated with poor outcome after mild TBI. By introducing alternatives to avoidant coping in the context of headache management, LCT may teach adaptive coping skills that generalize beyond headache management and improve other symptoms after mild TBI.

#### Sleep

Adequate sleep may play an important role in the evolution of headache disorders after TBI. Insomnia may be reported in the post-TBI population associated with headaches, evolving mood disturbance, or as one of the acute-onset symptoms of the head injury itself. TBI patients have reduced REM sleep (with increased slow-wave sleep) and produce lower levels of evening melatonin [136]. Obstructive sleep apnea, restless leg syndrome, and periodic limb movements of sleep are more common in the TBI population as well [137]. Sleep disturbance may contribute to the exacerbation of headache disorders and daytime cognitive complaints. It has also been postulated that insomnia reduces inhibitory pain control [138]. Concurrent use of benzodiazepines for indications such as anxiety, muscle spasm, or insomnia may also aggravate posttraumatic cognitive symptoms (even after discontinuation) as well as depression and should, therefore, be avoided when possible [139, 140].

#### Other lifestyle considerations

Self-medicating behaviors of concern may also involve use of caffeine, over-the-counter stimulants, marijuana, cocaine, alcohol, and other controlled or illicit substances. Alcohol has been associated with worsened cognitive performance in post-TBI populations and may interfere with the recovery process [141, 142].

While acute PTH resolve within a few weeks for the majority of individuals, some may go on to develop persistent PTH that can cause significant disability. Making matters more challenging for clinicians, there continues to be a lack of consensus regarding the management of persistent PTH, also due to the unmet need of randomized placebo-controlled trials. Current proper management of persistent PTH requires recognition of the primary headache type resembling by persistent PTH and tailoring pharmacologic and non-pharmacologic treatments to the individual patient. Based on the different presentations and the different treatment responses for each headache persistent PTH are entities apart from PTH.

Despite this, it is judicious to complete a thorough evaluation and exclude other secondary causes of headache and to provide each patient with an individualized and multidimensional treatment plan comprising lifestyle changes, psychological support, and pharmacological treatments. Because these headaches can be disabling and difficult to treat, further evidence-based approaches to this long-neglected field of research are needed to improve outcomes for affected patients.

## Conclusions

Persistent PTH is a disabling sequela with unknown pathophysiology and lack of specific treatments. It could be speculated that persistent PTH results in a migrainous loop, however, the relationship between persistent PTH and migraine is controversial and highly debated. These disorders share some phenotypic similarities, furthermore, the history of migraine is a risk factor for developing persistent PTH and migraine treatment is reported to be efficient in PTH patients.

In contrast, neuroimaging studies found different structural and functional brain changes, which suggest that these are distinct entities and have different mechanisms, and in PTH the brain changes could be reversible. Moreover, neurophysiological studies in PTH patients are sparse and fail to demonstrate specific features, while migraine patients present distinct neurophysiologic patterns.

Thus, despite some phenotypic similarities persistent PTH seems a distinct entity and high-quality studies are further required to investigate the pathophysiologic mechanisms of this secondary headache, in order to develop new targets for treatment and to prevent disability.

## Data Availability

All included references in the present review article are available on the Internet.

## Abbreviations

DLPFC: Dorsolateral prefrontal cortex
EEG: Electroencephalography
LCT: Learning to cope with triggers
SPG: Sphenopalatine ganglion
PPCS: Persistent post-concussion symptoms
PTH: Post-traumatic headache
TMS: Transcranial magnetic stimulation
TBI: Traumatic brain injury
VAS: Visual analog scale
